# Crystalline nanofiber photosensitizers with twisted dual-acceptors: high light harvesting and singlet oxygen quantum yield

**DOI:** 10.1093/nsr/nwaf524

**Published:** 2025-11-20

**Authors:** Xiaozhen Che, Chenglong Liao, Lishan Sun, Yanjun Gong, Hongwei Ji, Yanke Che, Ling Zang, Jin-Song Hu, Jincai Zhao

**Affiliations:** Key Laboratory of Photochemistry, CAS Research/Education Center for Excellence in Molecular Sciences, Institute of Chemistry, Chinese Academy of Sciences, Beijing 100190, China; University of Chinese Academy of Sciences, Beijing 100049, China; Key Laboratory of Photochemistry, CAS Research/Education Center for Excellence in Molecular Sciences, Institute of Chemistry, Chinese Academy of Sciences, Beijing 100190, China; University of Chinese Academy of Sciences, Beijing 100049, China; Key Laboratory of Photochemistry, CAS Research/Education Center for Excellence in Molecular Sciences, Institute of Chemistry, Chinese Academy of Sciences, Beijing 100190, China; University of Chinese Academy of Sciences, Beijing 100049, China; School of Chemistry and Chemical Engineering, Shandong University, Jinan 250100, China; Key Laboratory of Photochemistry, CAS Research/Education Center for Excellence in Molecular Sciences, Institute of Chemistry, Chinese Academy of Sciences, Beijing 100190, China; University of Chinese Academy of Sciences, Beijing 100049, China; Key Laboratory of Photochemistry, CAS Research/Education Center for Excellence in Molecular Sciences, Institute of Chemistry, Chinese Academy of Sciences, Beijing 100190, China; University of Chinese Academy of Sciences, Beijing 100049, China; Department of Materials Science and Engineering, University of Utah, Salt Lake City, UT 84112, USA; University of Chinese Academy of Sciences, Beijing 100049, China; Beijing National Laboratory for Molecular Sciences (BNLMS), CAS Key Laboratory of Molecular Nanostructure and Nanotechnology, Institute of Chemistry, Chinese Academy of Sciences, Beijing 100190, China; Key Laboratory of Photochemistry, CAS Research/Education Center for Excellence in Molecular Sciences, Institute of Chemistry, Chinese Academy of Sciences, Beijing 100190, China; University of Chinese Academy of Sciences, Beijing 100049, China

**Keywords:** photosensitizer, singlet oxygen, triplet state, photooxidation, nanofiber

## Abstract

The development of heavy-atom-free crystalline photosensitizers is highly favorable for practical applications due to their inherent advantages in robustness, facile post-reaction removal, and recyclability. However, achieving such systems with high molar absorptivity (>50 000 M⁻¹ cm⁻¹) and singlet oxygen quantum yields (>70%) remains a critical challenge, as these properties are typically compromised by intermolecular π-π interactions in molecular systems. Herein, we present a donor-acceptor (D-A) molecule featuring a uniquely twisted dual-acceptor backbone (D-A-A-D), achieving both high molar absorptivity and efficient singlet oxygen generation in monomeric solution. Critically, this connection topology facilitates the formation of crystalline nanofibers through CH/π and electrostatic interactions while effectively suppressing π-π stacking. The resulting crystalline nanofibers exhibit exceptional solid-state photophysical properties, including remarkably high molar absorptivity (ε = 53 400 M⁻¹ cm⁻¹) and singlet oxygen quantum yield (∼72%), surpassing even their monomeric forms. These synergistic attributes enable rapid, singlet oxygen-mediated aerobic photo-oxidation of organic substrates (e.g. benzylamines, sulfides). Furthermore, the nanofibers demonstrate excellent photostability and recyclability, retaining catalytic efficiency over at least five consecutive cycles. This work establishes crystalline photosensitizers as a new paradigm for integrating high molar absorptivity, exceptional singlet oxygen generation, and long-term structural durability.

## Teaser

Donor-acceptor (D-A) molecule featuring a unique twisting D-A-A-D backbone achieves exceptional light-harvesting capacity and efficient singlet oxygen generation in both solution and crystalline state.

## INTRODUCTION

Heavy-atom-free photosensitizers have garnered significant attention for their ability to circumvent drawbacks inherent in heavy-atom-containing analogues, such as dark toxicity, short triplet-state lifetimes, and poor photostability [1–20]. These advantages have propelled their adoption in diverse applications, including photodynamic therapy [3–12], wastewater treatment [13–15], and aerobic organic reactions [16–20]. Within this category, heterogeneous organic photosensitizers, particularly those in crystalline form, stand out due to their unique benefits. These include ease of post-reaction removal, recyclability, and highly ordered molecular architectures that enhance triplet exciton transport and photostability [15,19,20]. Such attributes render crystalline systems markedly advantageous over their homogeneous counterparts for practical applications. However, the rational design of crystalline organic photosensitizers concurrently achieving high singlet oxygen (^1^O_2_) quantum yields and robust molar absorptivity remains challenging. This stems from an inherent conflict: π-conjugated systems essential for strong light harvesting promote intense π-π interactions that quench both singlet and triplet states, substantially limiting ^1^O_2_ generation.

The molecular design of conventional photosensitizers, including tetrapyrrole-based systems (e.g. porphyrins) [4,7,19–26] and nonporphyrinoid frameworks such as boron dipyrromethene (BODIPY) [13,18,27–38], has historically prioritized maximizing ^1^O_2_ quantum yields in the monomeric form. This emphasis on photochemical efficiency has inadvertently overlooked critical aspects of controlled crystallization for solid-state photosensitizers, imposing practical limitations in applications requiring facile post-reaction removal, recyclability, and long-term photostability. Although structural modifications of existing photosensitizers offer a potential pathway to reconcile crystallinity with high ^1^O_2_ generation, introducing functional groups to enhance crystal packing often incurs laborious synthetic protocols while compromising photochemical performance. This inherent trade-off underscores the need for a paradigm shift in molecular design that simultaneously balances crystal control and functional efficacy.

To overcome these limitations, we propose a paradigm shift: the *de novo* design of photosensitizers using molecular scaffolds engineered for high crystallinity driven by non-π interactions (e.g. electrostatic forces). Our prior development of donor-acceptor (D-A) molecules featuring multiple donor/acceptor units within twisted backbones demonstrated their ability to maintain molecular photochemical properties in crystalline architectures [39–43]. This foundation enables the *de novo* crystalline photosensitizer design strategy presented here. Specifically, three key attributes underpin the utility of D-A systems: (1) twisted molecular backbones composed of multiple D and A units suppress undesirable intermolecular π-interactions, minimizing aggregation-induced quenching of excited states. (2) Electrostatic attraction drives programmable self-assembly, enabling the formation of crystalline architectures with tunable dimensionality. (3) Modular D-A scaffolds allow convenient modulation of donor/acceptor stoichiometry and spatial arrangement, tailoring intramolecular excited-state interactions to yield optoelectronic properties optimized for target applications. Despite these advantages, D-A systems with multiple interconnected units have yet to achieve high performance as photosensitizers in crystalline forms, though they excel as high-emission materials.

**Figure 1.**
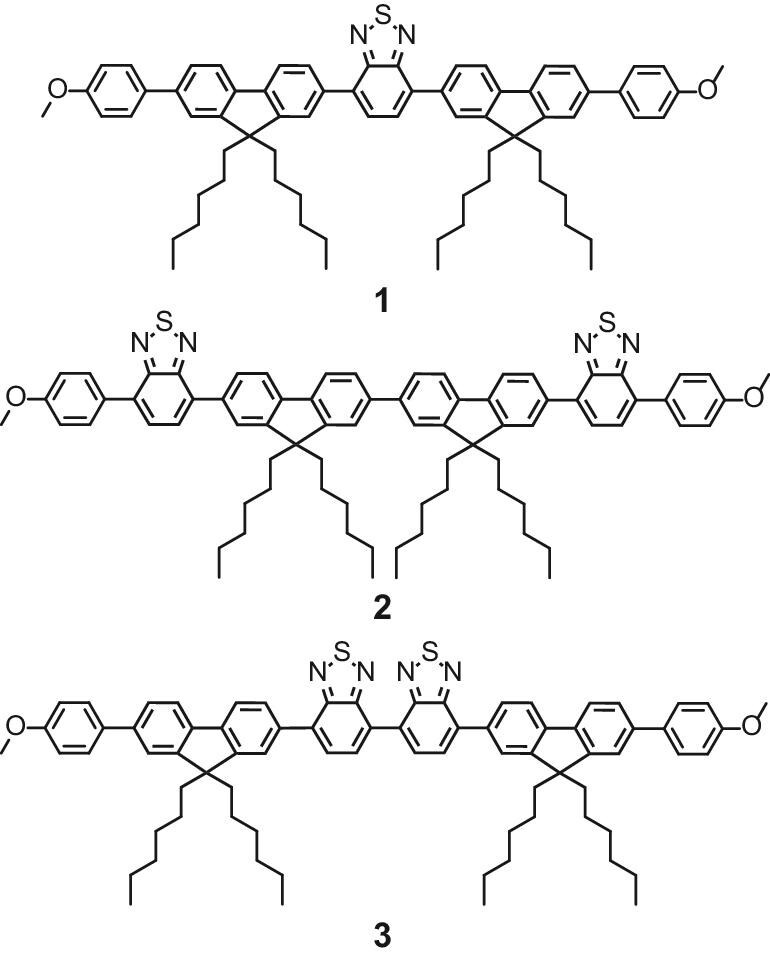
Molecular structures of **1**, **2**, and **3**.

A seminal study by Akkaya and coworkers demonstrated that orthogonal dimeric BODIPYs preserve two electronically decoupled chromophore cores, each enabling an independent HOMO→LUMO transition pathway [37]. This unique dimeric architecture introduces a mismatch between the S_1_ and S_0_ states and dramatically enhances intersystem crossing (ISC) efficiency to populate the triplet state. Inspired by this, we hypothesized that integrating two D-A subunits lacking heavy atoms into a twisted D-A-A-D backbone would: (1) retain sufficient electronic isolation to enhance triplet-state population and molar absorptivity while suppressing π-π interactions; (2) leverage electrostatic interactions to enable the formation of a crystalline structure. To validate this hypothesis, we designed molecule **3** with a D-A-A-D structure incorporating fluorene (donor) and benzothiadiazole (acceptor) units, aiming to assess whether this architecture achieves high molar absorptivity, exceptional singlet oxygen generation, and robust crystalline ordering. Control molecules **1** and **2** were also designed to provide comparative analysis, thereby validating the design rationale for molecule **3** (Fig. 1).

## RESULTS AND DISCUSSION

### Dual-acceptor design produces efficient intersystem crossing pathways

Molecule **1**, featuring a D-A-D structure with fluorene (donor) and benzothiadiazole (acceptor) units, was first developed as a reference for evaluating molar absorptivity and singlet oxygen generation. Subsequently, molecule **2** (with an A-D-D-A backbone) and molecule **3** (with a D-A-A-D structure) were synthesized to examine how the donor-acceptor connection topology influences these properties. The synthesis and characterization of molecules **1**–**3** are detailed in the Supporting Information (Figs S1–S6). Theoretical calculations revealed that all three D-A molecules exhibit twisted conformations between the donor and acceptor units (Fig. S7).

**Figure 2.**
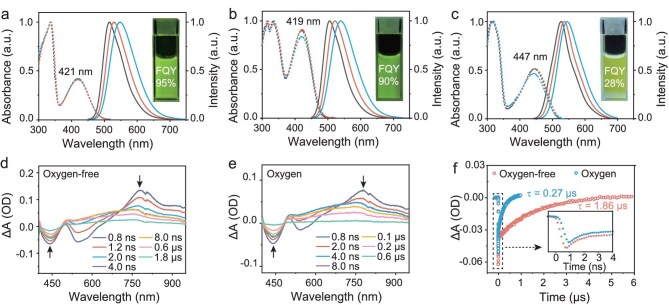
(a–c) Normalized absorption (dashed) and fluorescence spectra (solid) of molecules **1**, **2**, and **3** (5 μM) in cyclohexane (black), toluene (red), and chloroform (blue). (d, e) Transient absorption spectra of molecule **3** (50 μM) in toluene under oxygen-free and oxygenated conditions. (f) Time-resolved single-wavelength kinetics measured at 450 nm under oxygenated and oxygen-free conditions. Inset: zoom-in of decay kinetics within the initial 4 ns.

As shown in Fig. 2a, molecule **1** displayed characteristic charge-transfer (CT) absorption and emission bands. Notably, the CT absorption band remained relatively invariant across solvents, whereas the emission band exhibited a pronounced red shift with increasing solvent polarity. Quantitatively, molecule **1** exhibited exceptional fluorescence quantum yields (95% in toluene and chloroform), confirming radiative decay as the dominant excited-state dynamic and effectively suppressing intersystem crossing.

Similar to molecule **1**, molecule **2** featuring an A-D-D-A backbone structure exhibited characteristic CT absorption and emission bands (Fig. 2b). Notably, this system maintained exceptional fluorescence quantum yields (FQY ≈ 90%) in both toluene and chloroform, indicating that the dual-donor connectivity has minimal disruption on radiative decay pathways. Additionally, molecule **2** demonstrated a significantly enhanced molar absorptivity (61 200 M^−1^ cm^−1^) compared to molecule **1** (35 600 M^−1^ cm^−1^; Fig. S8), which we attribute to the simultaneous excitation of two D-A subunits within its symmetric structure. This finding is supported by calculations, which indicate that the electron density of the lowest unoccupied molecular orbital (LUMO) in molecule **2** is predominantly distributed across both acceptor units (Fig. S9). The molecular design featuring dual-donor connectivity suggests an effective strategy to enhance light absorption for implicants in advancing high-efficiency light-harvesting systems.

Similar to molecules **1** and **2**, molecule **3** featuring a D-A-A-D backbone structure exhibited characteristic CT absorption and emission bands (Fig. 2c). Similar to molecule **2**, molecule **3** demonstrated a marked increase in molar absorptivity (50 000 M^−1^ cm^−1^) relative to molecule **1** (35 600 M^−1^ cm^−1^; Fig. S8). This enhancement is attributed to the simultaneous excitation of two D-A sub-units within the structure, a mechanism corroborated by theoretical calculations of the molecule’s LUMO (Fig. S9). However, distinct from molecules **1** and **2**, molecule **3** exhibited substantially lower fluorescence quantum yields (FQY ≈ 28%) in both toluene and chloroform (Fig. 2c). Moreover, the maximum of both CT absorption and emission exhibited a markedly red-shifted profile compared to those of molecules **1** and **2**. These observations suggest that the dual-acceptor configuration introduces structural perturbations in the singlet excited state (S_1_), which facilitate non-radiative decay pathways.

To elucidate the specificity of non-radiative processes in molecule **3**, we measured transient absorption spectra of this molecule in toluene under oxygenated and oxygen-free conditions. As shown in Fig. 2d and e, a photobleaching band at 400–500 nm appeared immediately after excitation, accompanied by a concomitant rise in the 700–850 nm region. These observations are ascribed to the formation of S_1_. In the absence of oxygen, the photobleaching signal exhibited a rapid decay in the initial 4 ns, followed by a significantly slower decay phase that persisted until 1.8 μs (Fig. 2d, f). This biphasic behavior suggests an initial fast ISC from S_1_ to the triplet state (T_1_), with subsequent slow decay of the T_1_ population. In contrast, under oxygenated conditions, a similar S_1_→T_1_ transition occurred within 4 ns, afterwards the T_1_ decay was markedly accelerated (Fig. 2e, f), consistent with energy transfer to molecular oxygen to generate ^1^O_2_. Transient absorption spectra of **1** and **2** in toluene exhibited similar features: a photobleaching band (400–480 nm) and a rising absorption band (700–900 nm) emerged immediately after excitation, consistent with the formation of their S_1_. However, unlike the biphasic decay observed for **3** (attributed to S_1_→T_1_ intersystem crossing), the transient signals of **1** and **2** decayed monophasically to the ground state within ∼4 ns (Fig. S10). This rapid decay, observed under both aerobic and anaerobic conditions, confirms the absence of detectable T_1_ formation in **1** and **2**, aligning with their high fluorescence quantum yields. The formation of ^1^O_2_ for molecule **3** was further confirmed by electron paramagnetic resonance (EPR) spectroscopy using 2,2,6,6-tetramethylpiperidine (TEMP) as a spin trap (Fig. S11). Notably, the T_1_ quantum yield of molecule **3** was determined to be ∼60% based on the ratio of the photobleaching signal remaining at 8 ns to the initial signal.

Given the comparable S_1_–T_1_ energy gaps of molecules **1** and **3** (Fig. S12), the enhanced S_1_→T_1_ transition efficiency observed in molecule **3** cannot be simply attributed to a difference in the energy gap. Furthermore, although molecules **2** and **3** share identical donor-acceptor (D-A) subunits, their distinct connectivity topographies lead to divergent excited-state transition pathways. The efficient population of the T_1_ state in molecule **3** strongly implies that its excited-state dual-acceptor configuration promotes S_1_→T_1_ intersystem crossing, analogous to the behavior observed in orthogonal dimeric BODIPYs [37].

### Crystalline nanofibers of molecule 3

**Figure 3.**
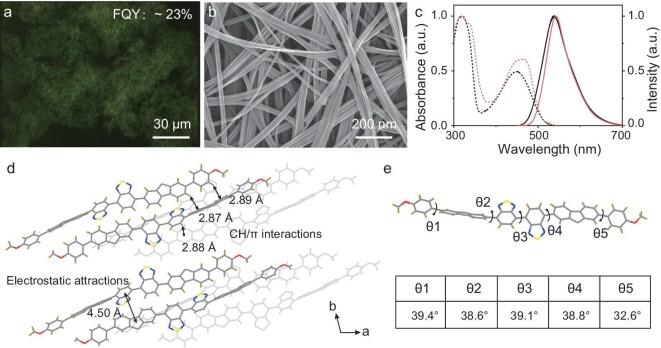
(a) Fluorescence-mode optical microscopic image of nanofibers of **3**. (b) SEM image of nanofibers of **3**. (c) Normalized absorption (dashed) and fluorescence spectra (solid) of nanofibers of **3** (red) cast on a quartz slide and molecule **3** (black) in toluene (5 μM). (d) Molecular packing of **3** along different directions, where the dominant intermolecular forces driving crystal growth were identified as CH/π and electrostatic attractions. (e) Significantly twisted dihedral angles between D and A units in the molecular backbone of **3**.

Following the observation of high molar extinction coefficients and ^1^O_2_ quantum yields for molecule **3** in solution, we sought to determine whether it could self-assemble into crystalline nanostructures without compromising these photophysical properties. Aggregates were generated by injecting 0.2 mL of a chloroform solution of molecule **3** (1 mg/mL) into 2 mL of methanol within a 4 mL vial, followed by aging for 2 h. The aggregates precipitated after 10 min and settled at the vial bottom, emitting green fluorescence with FYQ of 23% (Fig. 3a). Scanning electron microscopy (SEM) revealed the formation of nanofibers with lengths of several micrometers and widths ranging from 30 to 60 nm (Fig. 3b and Fig. S13). The crystallinity of the nanofibers was established via powder X-ray diffraction (PXRD), which displayed distinct diffraction peaks (Fig. S14). To further validate the crystalline nature of the nanofibers, we conducted PXRD analysis on larger nanofibers (widths: 200–450 nm) fabricated via seeded self-assembly. These enlarged structures exhibited identical Bragg peak positions, but significantly sharper reflections compared to the original nanofibers (Fig. S14), confirming retention of the same lattice structure. The thermal stability of the nanofibers was confirmed by thermogravimetric analysis (TGA), which demonstrated structural integrity retention up to 360°C without significant loss of mass (Fig. S15). Notably, the absorption and fluorescence spectra of the nanofibers exhibited a modest redshift relative to the solution-phase molecule **3** (Fig. 3c). This phenomenon is attributed to either weak electronic coupling between adjacent molecules within the crystalline lattice or slight backbone twisting.

To gain a deep insight into the conformation and orientation of molecule **3** within nanofibers, we prepared larger fiber crystals via a living seeded self-assembly method (see the Supporting Information for details), which was suitable for single-crystal X-ray diffraction (SCXRD) analysis. The single crystal fibers crystallized in the triclinic P-1 space group (Table S1). The single crystal fibers and nanofibers share identical primary PXRD peaks (Fig. S14), confirming that the nanofibers adopt the same triclinic P-1 lattice as the single crystal. As illustrated in Fig. 3d and e, and Fig. S16, the benzothiadiazole-fluorene units adopt a significantly twisted conformation, with large dihedral angles effectively suppressing π-π interactions between adjacent molecules. Instead, the dominant intermolecular forces driving the assembly were identified as CH/π interactions and electrostatic attractions between D and A groups (Fig. 3d) [39–41], which direct the anisotropic growth of nanofibers along the b-axis (the long axis of nanofibers). In contrast, weak hydrophobic interactions along the remaining two axes govern lateral growth, defining the thickness of the nanostructures. This twisted conformation and lack of π-stacking align well with the observed optical properties: the absorption spectra of the nanofibers closely resemble those of molecule **3** in solution (Fig. 3c). This similarity further implies comparable molar absorption coefficients between the solid-state nanofibers and molecularly dispersed **3**. Consistent with this, the molar absorptivity of nanofibers of **3** dispersed in ethanol was measured as 53 400 M^−1^ cm^−1^ (Figs S17 and S18), corroborating the retention of solution-like electronic transitions in the assembled nanostructures.

### Quantum yield determination of ^1^O_2_ for crystalline nanofibers

**Figure 4.**
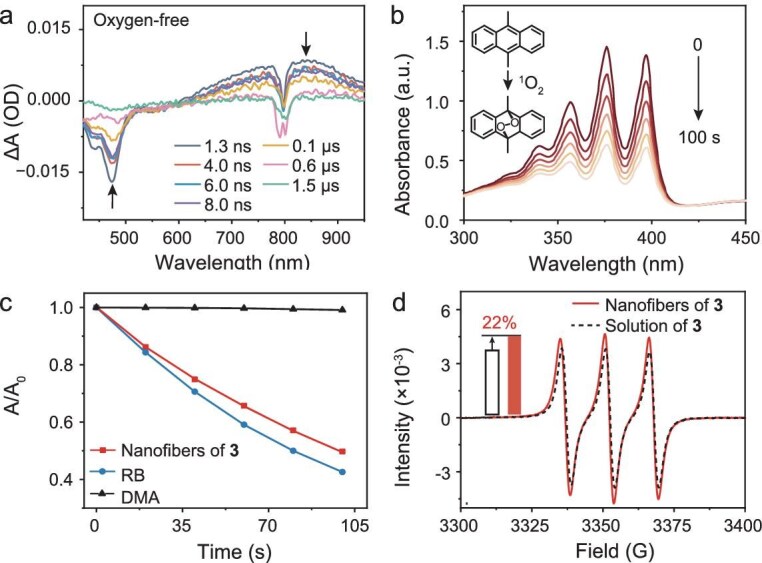
(a) Transient absorption spectra of crystalline nanofibers of **3** (0.12 mg in 2 mL of ethanol) under oxygen-free conditions. Note: signals between 765–815 nm arise from the instrument-generated second harmonic artifact of the excitation pulse (2ω_ex_ = 800 nm). (b) Time-dependent absorption spectra of DMA (300 μM) in 2 mL ethanol containing nanofibers of **3** (0.01 mg) upon 470 nm LED irradiation (30 mW/cm^2^). Inset: ^1^O_2_-mediated oxidation of DMA reaction. (c) The absorbance change of DMA (300 μM in 2 mL ethanol) recorded at 376 nm as a function of irradiation time without photosensitizers and in the presence of nanofibers of **3** (0.01 mg in 2 mL of ethanol) or RB (30 μM). (d) EPR spectra of nanofibers of **3** in ethanol (0.5 mg/mL; red) and molecule **3** in toluene (0.5 mg/mL; black) obtained after 5 min of 470 nm LED irradiation (30 mW/cm^2^), showing the enhanced ^1^O_2_ yield of nanofibers of **3** relative to the monomeric form of **3** in toluene under identical conditions (inset).

We next quantified the T_1_ quantum yield of crystalline nanofibers of **3** using transient absorption spectroscopy. Under oxygen-free conditions, excitation of nanofibers of **3** suspended in ethanol produced an immediate photobleaching band (400–500 nm) and a concomitant rise in excited-state absorption (ESA) at 650–900 nm (Fig. 4a), characteristic of the S_1_. Beyond 6 ns, the photobleaching signal decayed biphasically, exhibiting a slow decay phase persisting to 1.5 μs (Fig. 4a). Analogous to monomeric **3**, this biphasic kinetics indicates rapid S_1_→T_1_ ISC followed by slow T_1_ decay. The T_1_ quantum yield was calculated as ∼72% based on the residual-to-initial photobleaching signal ratio at 8 ns. To further validate the ^1^O_2_ quantum yield of nanofibers of **3**, we employed chemical trapping to quantify ^1^O_2_ generation from crystalline nanofibers of **3** [42–44]. Specifically, 9,10-dimethylanthracene (DMA) served as the ^1^O_2_ probe, while Rose Bengal (RB; Φ_RB_ = 0.86 in ethanol) acted as the reference photosensitizer. As shown in Fig. 4b and Fig. S19, irradiation of nanofibers of **3** or RB with a 470 nm LED (30 mW/cm^2^) induced a time-dependent decrease in DMA absorbance at 376 nm, attributed to ^1^O_2_-mediated oxidation of DMA. By combining the absorbance values of nanofibers of **3** and RB at 470 nm (Fig. S20), the ^1^O_2_ quantum yield of crystalline nanofibers of **3** was calculated as 75% (Fig. 4c; see Supporting Information for detailed calculations), which is consistent with that (72%) quantified based on transient absorption results. Notably, this value (75%) exceeds that of molecularly dissolved **3** in toluene (60%). EPR analysis further revealed that nanofibers of **3** in ethanol (0.5 mg/mL, 200 μL) exhibited a 1.22-fold increase in the ^1^O_2_-TEMP signal intensity compared to molecular dissolved **3** in toluene under identical experimental conditions (Fig. 4d). This enhancement aligns with the direct proportionality between the^1^O_2_ quantum yields of the crystalline nanofibers and the molecularly dissolved species in toluene. The observed boost in ^1^O_2_ quantum yield is attributed to the restricted molecular motion of **3** within the nanofibers, which reduces competitive non-radiative pathways and thereby promotes more efficient ISC compared to the freely rotating molecules in solution. This restriction effect is evidenced by the narrowed fluorescence spectrum of crystalline nanofibers relative to monomeric **3** (Fig. 3c). In contrast to nanofibers of **3**, aggregates of molecules **1** and **2** (Fig. S21) exhibited no detectable generation of ^1^O_2_ (Fig. S22), consistent with their monomeric counterparts. This absence of ^1^O_2_ production was further supported by the lack of changes in DMA absorption spectra and negligible ^1^O_2_-TEMP signal intensity in the presence of aggregates of **1** and **2** (Fig. S22).

### Photooxidation mediated by ^1^O_2_

Owing to their high molar absorptivity and ^1^O_2_ quantum yield—a combination rare for solid materials (Table S2)—crystalline nanofibers of **3** were expected to serve as efficient heterogeneous photosensitizers for photooxidation reactions, providing inherent recovery and recyclability advantages. To evaluate the practical utility of nanofibers of **3**, we investigated their photosensitive activity in the oxidation of two representative organic substrates: benzylamines and sulfides. These standard substrates were chosen primarily due to their well-established reaction mechanism with ^1^O_2_, as presented in Fig. S23 [19,24].

**Figure 5.**
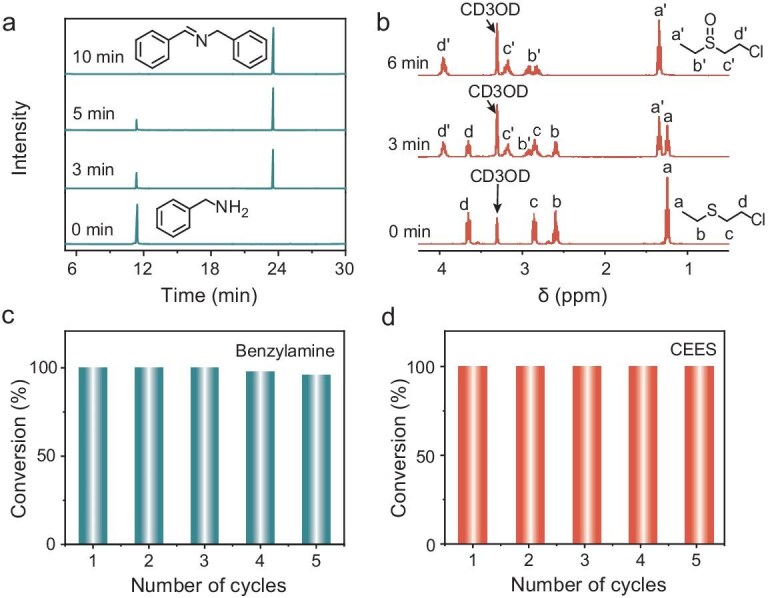
(a) GC analysis of the photosensitized oxidative coupling of benzylamine (50 mM) in acetonitrile containing 0.5 mol% nanofibers of **3** under 470 nm LED irradiation (30 mW/cm^2^). (b) ^1^H NMR analysis of the oxidation of CEES (50 mM) in CD_3_OD containing 1 mol% nanofibers of **3** under 470 nm LED irradiation (100 mW/cm^2^). (c) Five cycles of the oxidative coupling of benzylamines as performed in (a). (d) Five cycles of the oxidation of CEES as performed in (b).

The photooxidation reaction of benzylamines (50 mM) was conducted in acetonitrile using 0.5 mol% nanofibers of **3** relative to benzylamine. The reaction was initiated by LED irradiation at 470 nm (30 mW/cm^2^) under an oxygen atmosphere. GC-MS analysis revealed that the coupling of benzylamine to N-benzylidenebenzylamine reached completion within 10 min of irradiation, as illustrated in Fig. 5a and Fig. S24. Furthermore, the reaction selectivity was confirmed to exceed 99% through GC-MS and ¹H NMR analyses (Figs S24 and S25). Similar results were obtained for other benzylamines under identical conditions (Table 1 and Figs S26–S29). A control experiment was performed using 1,4-diazabicyclo[2.2.2]octane (DABCO) as a ^1^O_2_ quencher. GC analysis showed that product formation was completely suppressed upon addition of DABCO, strongly supporting a ^1^O_2_-mediated mechanism (Fig. S30). Notably, after the coupling reaction, the nanofiber fully settled at the bottom of the flask within 20 min (Fig. S31). Owing to its retained emissivity as mentioned above (∼23%), the photosensitizer remained easily visible under irradiation, thereby facilitating post-reaction recovery and recyclability.

**Table 1.** Photosensitive performance of nanofibers of **3** in coupling of benzylamines.^a^

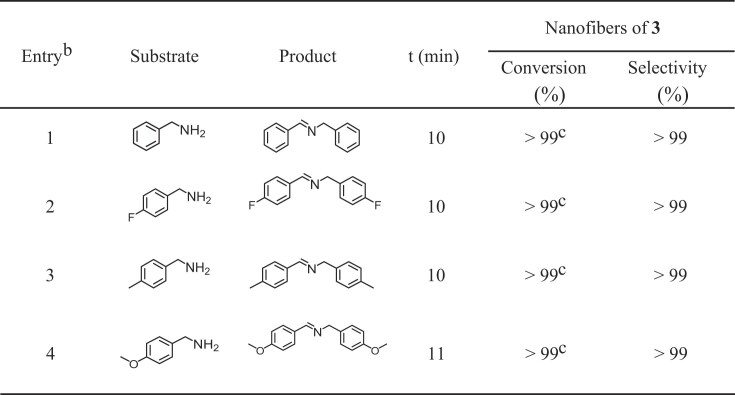

a Reaction conditions: 50 mM benzylamines, 0.5 mol% nanofibers of **3**, 2 mL acetonitrile, 470 nm LED irradiation (30 mW/cm^2^), O_2_ atmosphere (1 amp), and room temperature. ^b^Conversion determined by GC-MS; selectivity calculated by GC-MS and ^1^H NMR analysis. ^c^No detectable unreacted starting materials within the sensitivity limits of our analytical methods.

The photooxidation of 2-chloroethyl ethyl sulfide (CEES) and thioanisole was carried out in deuterated methanol (CD_3_OD) containing 1 mol% nanofibers of **3** (relative to the substrate, 50 mM) under 470 nm LED irradiation (100 mW/cm^2^). For CEES, ^1^H NMR analysis revealed complete conversion to 2-chloroethyl ethyl sulfoxide (CEESO) within 6 min (Fig. 5b), with a selectivity exceeding 99% (Table S3 and Fig. S32). Similarly, GC-MS analysis confirmed full conversion of thioanisole to methyl phenyl sulfoxide within 25 min, demonstrating 94% selectivity as corroborated by both GC-MS and ^1^H NMR (Table S3, Figs S33 and S34). Consistent with previous observations, the nanofiber settled entirely at the bottom of the flask post-reaction within 20 min and remained visible under irradiation owing to its retained emissivity (Fig. S31).

The above results demonstrate that nanofibers of **3** function as high-performance heterogeneous photosensitizers for photooxidation reactions. To evaluate their reusability, we tested the catalysis cycles of nanofibers of **3** in the photooxidation of benzylamine and CEES. As shown in Fig. 5c and d, the nanofibers maintained stable catalytic activity over five consecutive cycles for both substrates. MALDI-MS, XRD, and FT-IR analyses confirmed the structural and functional robustness of nanofibers of **3** after recycling. The molecular mass (MALDI-MS), characteristic functional groups (FT-IR), and crystalline lattice (XRD) remained unaltered, as demonstrated in Figs S35 and S36. Critically, the emissive nanofibers retained their macroscopic fibrous morphology throughout the cycles, as evidenced by their rapid settling to the bottom of the reaction flask after each use. This behavior enabled facile separation of the supernatant and efficient reintroduction of fresh reactants for subsequent cycles, underscoring their practical utility in flow or batch processes.

## CONCLUSIONS

In summary, we report the development of a heavy-atom-free D-A molecular system featuring a twisted dual-acceptor backbone (D-A-A-D) that achieves both high molar absorptivity and efficient singlet oxygen (^1^O_2_) generation in solution. Comparative studies with D-A-D and A-D-D-A analogues demonstrate that the dual-acceptor configuration enhances intersystem crossing (ISC) from the S_1_ to T_1_ state, as corroborated by transient absorption spectroscopy. Critically, the twisted architecture promotes CH/π and electrostatic interactions over π-π stacking, driving the formation of crystalline nanofibers. These nanofibers exhibit high molar absorptivity (53 400 M^−1^ cm^−1^) and efficient ^1^O_2_ generation (∼72%), enabling rapid, visible-light-driven photooxidation of benzylamines and sulfides under mild conditions (complete conversion within minutes). Furthermore, the nanofibers retain their macroscopic fibrous morphology, allowing easy recovery via sedimentation and consistent photosensitizing performance over multiple reaction cycles. This work establishes crystalline photosensitizers as a new design paradigm for unifying high molar absorptivity, efficient singlet oxygen generation, and long-term structural durability.

## METHODS

See details in the online Supplementary data.

## Supplementary Material

nwaf524_Supplemental_Files
