# Supplementary material for: Crystalline nanofiber photosensitizers with twisted dual-acceptors: high light harvesting and singlet oxygen quantum yield
Source: Natl Sci Rev. 2025 Nov 20;13(1):nwaf524. doi: 10.1093/nsr/nwaf524 (PMC12796797; doi:10.1093/nsr/nwaf524)
Supplement: nwaf524_Supplemental_Files [file nwaf524_supplemental_files.zip › Teaser text.docx]

Donor-acceptor (D-A) molecule featuring a unique twisting D-A-A-D backbone achieves exceptional light-harvesting capacity and efficient singlet oxygen generation in both solution and crystalline state.
